# Green Synthesis of Gold Nanoparticles with Good Photothermal Properties and Antibacterial Activity from Black Corncob Extract

**DOI:** 10.3390/nano16110646

**Published:** 2026-05-22

**Authors:** Yingwei Li, Fangsu Liu, Zhiguo Liu

**Affiliations:** 1 College of Food and Health, Northeast Forestry University, Harbin 150040, China; 15689396952@163.com; 2 Aulin College, Northeast Forestry University, Harbin 150040, China; 3 College of Chemistry, Chemical Engineering and Resource Utilization, Northeast Forestry University, Harbin 150040, China; 13863906435@163.com; 4 Key Laboratory of Forest Plant Ecology, Ministry of Education, Northeast Forestry University, Harbin 150040, China; 5 Engineering Research Center of Forest Bio-Preparation, Ministry of Education, Northeast Forestry University, Harbin 150040, China; 6 Heilongjiang Provincial Key Laboratory of Ecological Utilization of Forestry-Based Active Substances, Northeast Forestry University, Harbin 150040, China

**Keywords:** green synthesis, gold nanoparticles, black corncob, photothermal, antibacterial activity

## Abstract

Green synthesis of gold nanoparticles is an effective approach to create biocompatible nanomaterials. In this study, gold nanoparticles (BC-AuNPs) were prepared by reducing chloroauric acid with black corncob (BC) extract at relatively low temperatures. The optimal preparation conditions were obtained through a single-factor experiment, which included 5 mL of black corncob extract and 0.12 mL of 3% HAuCl_4_ solution at a pH of 5.0, and the reaction was carried out at 50 °C in a water bath for 3 h. The prepared BC-AuNPs were characterized by ultraviolet–visible (UV-Vis) spectroscopy, Fourier-transform infrared (FTIR) analysis, transmission electron microscopy (TEM), high-resolution transmission electron microscopy (HRTEM), scanning electron microscopy (SEM), dynamic light scattering (DLS), and Zeta-potential measurement, which showed that they were dispersed spherical particles with an average size of approximately 23.0 nm and their surfaces were covered with various black corncob active components. The photothermal performance test indicated a good photothermal effect with a conversion efficiency of 41.3%. Antibacterial experiments revealed that BC-AuNPs had excellent antibacterial activity. The minimum inhibitory concentrations (MICs) for *E. coli* and *Salmonella* were 25.00 and 50.00 µg/mL, respectively. Overall, this study proved a potential application for gold nanoparticles in photothermal antibacterial fields.

## 1. Introduction

Gold nanoparticles have become one of the most studied nanomaterials in recent years due to their antioxidant properties, stability, and good biocompatibility. The unique properties of gold nanoparticles make them widely used in various fields such as photothermal therapy [1], biosensors [2], catalysis [3], antibacterial applications [4], drug and gene delivery [5], and imaging technologies [6]. Previous studies have shown that gold-based nanostructures with different morphologies all exhibit antibacterial mechanisms, including increased ROS generation, PTT, PDT, and physical interactions [7]. Antibacterial agents derived from gold nanoparticles have relatively low resistance [8]. Furthermore, gold nanomaterials exhibit local surface plasmon resonance, which can generate heat when exposed to resonant light. This unique optical property gives them great potential for photothermal therapy due to their short treatment time and low side effects [9,10]. Lopatynskyi et al. [11] proved that solid and hollow gold nanostructures represented by spherical nanoparticles, nanocapsules, and nanocages in colloidal solutions were an effective photothermal treatment agent.

Traditional approaches for preparing gold nanoparticles frequently involve toxic chemicals, generate hazardous by-products, and involve high costs and substantial energy input, raising doubts about environmental sustainability and potential risks to human health [12]. Using plant extracts for gold nanomaterial synthesis does not require the complex maintenance of microbial cultures compared with the use of microorganisms such as fungi or bacteria and is therefore regarded as a great green synthesis approach [13]. The plant extracts contain a large number of biological molecules, such as alkaloids, flavonoids, terpenoids, and phenolic compounds, which can function as reducing agents and stabilizers and can effectively prevent the aggregation of nanoparticles and control their size and morphology [14]. Jiao et al. [15] verified that green-synthesized gold nanoparticles could lead to a reduction in the activity of malignant gastric cells and possessed anti-gastric cancer efficacy. The medicinal plant *Equisetum diffusum* D. was surface-functionalized to enhance the biological activity of gold nanoparticles. As a result, the synthesized ED-AuNPs exhibited remarkable activities in antibacterial, anticancer, antidiabetic, and antioxidant fields and demonstrated potential as a multifunctional material [16]. Furthermore, green gold nanoparticles synthesized from plant extracts exhibit particularly outstanding antibacterial properties. Gold nanoparticles green-synthesized based on an extract from *Carissa macrocarpa* fruits inhibited the growth of both Gram-positive and Gram-negative bacterial strains [17]. Gold nanoparticles synthesized from an extract from *Tagetes patula* L. flowers exhibited antibacterial activity against plant and human pathogens and showed larger inhibition zones than traditional antibiotics [18]. Gold nanoparticles prepared from extracts of geranium leaves and castor seeds also exhibited antibacterial activity, significantly damaging bacterial cell walls, as verified by SEM imaging [19,20].

Green synthesis of gold nanomaterials, used as effective antibacterial agents, is considered one of the solutions for antibacterial therapies [21]. Antimicrobial resistance (AMR) has become a serious global health issue and is listed by the WHO among the top 10 public health threats of the 21st century. Research has shown that among the 4.95 million deaths attributable to bacteria worldwide, 26% were directly attributable to AMR [22]. Therefore, the development of gold nanomaterials with excellent antibacterial performance can effectively address bacterial resistance.

In this study, we offer an effective synthesis method: employing a black corncob extract as both a reducing agent and a stabilizer for the green synthesis of gold nanoparticles. To the best of our knowledge, the application of black corncob extract in the synthesis of gold nanoparticles has not yet been systematically explored, and the mechanism by which its active ingredients facilitate nanoparticle formation remains unclear. The key role of the active components of black corncob in the formation of nanoparticles was revealed through a single-factor experimental system to optimize the synthesis parameters (extract liquid addition amount, chloroauric acid addition amount, pH, and reaction time) and by using multi-dimensional characterization methods such as liquid chromatography–mass spectrometry (LC-MS), UV-Vis, FTIR, TEM, HRTEM, SEM, DLS, and Z-potential. Furthermore, the photothermal properties of the synthesized BC-AuNPs were evaluated, and antibacterial activity was verified for common clinical multidrug-resistant bacteria (*A. baumannii*, *E. coli*, and *Salmonella*). This study not only provides a new paradigm for the green synthesis of gold nanoparticles but also offers new ideas for the design of novel photothermal antibacterial materials in biomedicine.

## 2. Materials and Methods

### 2.1. Experimental Materials

The black corncob powder was self-prepared in the laboratory using fresh corncobs purchased from Harbin, China. Chloroauric acid (AuCl_3_·HCl·4H_2_O), of an analytical purity ≥ 99.8%, was purchased from Sinopharm Chemical Reagent Co., Ltd., Shanghai, China. NH_3_·H_2_O, of an analytical purity ≥ 99.8%, was purchased from Tianjin Fuyu Chemical Co., Ltd., Tianjin, China. The experimental water was ultra-pure water (resistivity ≥ 18.2 MΩ·cm, prepared by the Milli-Q system, Merck KGaA, Darmstadt, Germany).

### 2.2. Preparation of Black Corncob Extract

First, 3.00 g of black corncob powder was weighed and placed in a conical flask. Next, 75% ethanol was added at a liquid-to-solid ratio of 1:80 g/mL. Then, microwave extraction was performed at 50 °C for 15 min. After extraction, we waited for the temperature to drop to room temperature. The supernatant of the black corncob extract was obtained by centrifuging at 10,000 rpm for 10 min and it was stored in the dark at 5 °C for future use.

### 2.3. LC-MS Analysis of Chemical Components in Black Corncob Extract

The prepared black corncob extract solution was diluted with acetonitrile. The sample solution was filtered through a 0.22 μm filter membrane and injected into an LC-MS (Thermo ScientificTM Q ExactiveTM Focus, Thermo Fisher Scientific, Waltham, MA, USA) with an electrospray ionization source. The data were collected in positive ionization mode. Based on the retention time, the mass-to-charge ratio of the product ion, and the fragmentation information of MS/MS, the active components in the extract were qualitatively characterized.

(1) UPLC operating parameters: The column was maintained at a constant temperature of 40 °C, and the sample injection volume was set to 5 μL. The mobile phase consisted of two components: phase A was an aqueous solution containing 0.1% formic acid, and phase B was acetonitrile. The flow rate of the mobile phase was 0.35 mL/min. A gradient elution program was adopted with the following specific procedures: at 0 to 1.00 min, phase B accounted for 2%; at 1.00 to 9.00 min, phase B was linearly increased from 2% to 98%; at 9.00 to 12.00 min, phase B was kept stable at 98%; at 12.00 to 12.10 min, phase B was rapidly decreased from 98% to 2%; and at 12.10 to 15.00 min, phase B remained at 2% to equilibrate the column.

(2) MS/MS detection conditions: A heated electrospray ionization source was employed as the ion source, with the detailed parameters configured as follows: the sheath gas (N_2_) flow rate was 40 units, the auxiliary gas (N_2_) flow rate was 10 units, and the purge gas (N_2_) flow rate was set to 0 units. The spray voltage was adjusted to 3.0 kV, the ion transport tube temperature was 320 °C, the S-lens RF level was 60, and the atomization heater temperature was 350 °C. Data acquisition was performed in Full MS-ddMS_2_ mode, with positive and negative ion switching scanning. The acquisition time was consistent with that of the UPLC system. For the specific acquisition settings, high-resolution full-scan primary mass spectrometry (MS) data were collected at a resolution of 70,000, covering a mass-to-charge ratio (*m*/*z*) range of 80 to 1200. Secondary mass spectrometry (MS_2_) data were acquired in Top1 mode at a resolution of 17,500, with collision energies set at 20, 40, and 60 eV, respectively.

### 2.4. Preparation of BC-AuNPs

To prepare the BC-AuNPs, 0.8 g of solid chloroauric acid was weighed and dissolved in 26.7 mL of ultra-pure water. It was stored in a dark place at 5 °C before use.

The black corncob extract was used as a reducing agent and stabilizer, and BC-AuNPs were prepared under a water bath at 50 °C. A typical experiment was as follows: 5 mL of black corncob extract and 0.12 mL of 3% HAuCl_4_ solution were mixed and transferred to a centrifuge tube. The solution was diluted with ultra-pure water to a total volume of 10 mL and the pH was adjusted to 5.0 using NH_3_·H_2_O. The solution was reacted at 50 °C in a water bath for 3 h. After the reaction was completed, the synthesized product, a wine-red colloidal solution, was obtained.

The synthesis procedure was optimized via single-factor experiments by considering the morphology, distribution, and yield of BC-AuNPs. A single-factor experiment was conducted to investigate the effects of the amount of black corncob extract added, the amount of 3% HAuCl_4_ solution added, different pH conditions, and reaction time. Under the condition that all other factors remained constant, each parameter was changed only once. The specific settings are given in Table 1.

Based on the results of the above single-factor experiments, the optimal process for preparing BC-AuNPs under 50 °C water bath conditions was determined. The synthetic products were collected under the optimal process conditions. The samples were then stored in a dark place at 5 °C before use.

### 2.5. Structural Characterization

The UV-Vis absorption spectrum of the BC-AuNPs was measured using a IMPLEN NP80 UV-Vis spectrophotometer (Implen GmbH, Munich, Germany). The surface plasmon resonance characteristics of the diluted BC-AuNPs (diluted three-fold) were determined with a scanning range of 350–900 nm and ultra-pure water as the blank control. The UV-Vis absorption spectrum of the diluted black corncob extract (diluted three-fold) was also measured for comparison. FTIR analysis was conducted using a Shimadzu IR Affinity-1 spectrometer (Shimadzu Corporation, Kyoto, Japan). The potassium bromide pellet method was employed. Approximately 160 mg of dry potassium bromide powder and BC-AuNPs were mixed, ground, and pressed as a tablet for measurement. TEM and HRTEM were performed using a JEM-2100 transmission electron microscope, JEOL Ltd., Tokyo, Japan (with an accelerating voltage of 200 kV). A volume of 5 µL of the BC-AuNP sample solution was dropped onto a carbon-coated copper grid and allowed to dry naturally at room temperature for high-resolution imaging. SEM was conducted using a JSM-7610FPlus field emission scanning electron microscope, JEOL Ltd., Tokyo, Japan (with a high voltage of 5 kV). A volume of 5 µL of the BC-AuNP sample solution was dropped onto a carbon-coated copper net and then left to dry at room temperature. DLS and Z-potential measurements were conducted using the Microtrac Dynamic Light Scattering Particle Size Analyzer (Microtrac MRB, York, PA, USA). The test was carried out using ultra-pure water as the dispersion medium. The sample cell temperature was controlled at 23 °C. Each test lasted for 30 s. Before the test, the sample was diluted two-fold to meet the instrument’s load index requirements.

### 2.6. Determination of Photothermal Properties

Under the irradiation of an 808 nm laser (with a power density of 2.1 W/cm^2^), the temperature changes in the BC-AuNP solutions with concentrations of 0.2 mg/mL, 0.1 mg/mL, 0.05 mg/mL, 0.025 mg/mL, 0.0125 mg/mL, and 0.00625 mg/mL were monitored using an infrared thermal imager (Testo865, German Instrument International Trade (Shanghai) Co., Ltd., Shanghai, China). The irradiation time was 10 min in total. Temperature data was recorded every 30 s and thermal images were captured every 2 min; using ultra-pure water as a blank control, all experiments were repeated three times.

A photothermal cycling experiment was conducted on a 0.2 mg/mL BC-AuNP solution. Each cycle consisted of two stages: first, the laser was turned on to irradiate the BC-AuNP solution for 10 min and then the light source was turned off to allow the system to cool naturally for another 10 min. During this process, an infrared thermal imager was used to continuously monitor and record the temperature changes in the solution every 30 s. A total of 5 cycles were measured.

As for photothermal conversion efficiency determination, a photothermal cycling test was conducted on a 0.2 mg/mL BC-AuNP solution. The cycle consisted of a 10 min irradiation period followed by a natural cooling period. The temperature changes in the solution were continuously monitored using an infrared thermal imager at intervals of 30 s. Then, based on the obtained data, the photothermal conversion efficiency was calculated. The calculation formula was as follows [23,24]:

(1) $$\eta \;=\;\frac{\mathrm{hS}(T_{\mathrm{Max}}\;-\;T_{\mathrm{Sur}})\;-{\;Q}_{\mathrm{Dis}}}{I(1\;-\;10^{-A})}$$

(2) $$\mathrm{hS}=\frac{\mathrm{mc}}{\tau }$$

(3) $$\tau =-\frac{\mathrm{dt}}{\mathrm{dIn}\theta }$$

(4) $$\theta =\frac{T_{t,c}\;-\;T_{\min,c}}{△T_{s,c}}$$

In the formula “η” is the photothermal conversion efficiency; “$T_{\mathrm{Max}}$” represents the maximum temperature reached by the BC-AuNP solution within 10 min; “$T_{\mathrm{Sur}}$” refers to the ambient temperature of the photothermal agent; “A” represents the absorbance of the photothermal agent at 808 nm; “I” represents the laser power of the 808 nm light; “c” is the specific heat capacity of water (4.2 × 10^3^ J/(kg·°C)); “m” represents the mass of 1 mL of the photothermal agent; “τ” is the slope of the graph representing the natural logarithmic relationship between cooling time and temperature; “t” is the time within the cooling curve; “$△T_{s,c}$” is the temperature change value of the BC-AuNP solution during the cooling process; “$T_{\min,c}$” is the final temperature in the cooling curve; and “$T_{t,c}$” is the solution temperature in the cooling curves at different times.

### 2.7. Antibacterial Experiment

Three types of Gram-negative bacteria, namely *A. baumannii*, *E. coli* and *Salmonella* were selected as the test strains for the study of the antibacterial properties of the BC-AuNP solution. Antibacterial activity was investigated using the inhibition-zone experiment method. The experimental samples for the antibacterial experiment were 0.2 mg/mL BC-AuNP solution, 0.01% penicillin solution, black corncob extract, and 3% HAuCl_4_ solution. Among them, the latter three were the control samples.

The bacteria used were taken out of the refrigerator and activated by shaking at 37 °C for 30 min. To 5 mL of the broth, 5 µL of the bacterial solution diluted 1000 times was added. Next, 250 µL of *A. baumannii* was added to the agar medium, it was spread evenly using a spreader, and finally sterilized forceps were used to place four dry filter paper sheets (20 µL of 0.01% penicillin solution, black corncob extract, 3% HAuCl_4_ solution, and 0.2 mg/mL BC-AuNP solution, respectively, were dropped on the four filter paper disks) into the bacterial plate to allow the solution to be fully absorbed. The same operation was carried out by adding 250 µL of *E. coli* and *Salmonella* to the agar medium, respectively. All the plates were sealed and incubated upside down at a temperature of 37 °C for 24 to 48 h. The growth of the colonies was observed at the edge of the filter paper. If colony growth was hindered, a circular inhibition zone would form between the edge of the filter paper and the colony. This area was photographed for analysis.

The MIC was determined using the two-fold dilution method. A volume of 100 µL of nutrient broth and 100 µL of the sample were mixed evenly in the first well of a 96-well plate. Then 100 µL of the liquid was transferred to the next well, and 100 µL of nutrient broth medium was added. This process was repeated until the last well, and 100 µL of the liquid was discarded. Subsequently, 10 µL of a 1 × 10^6^ CFU/mL bacterial suspension was added to each well. After the plate was covered, the same cultivation conditions as the inhibition-zone experiment were applied. The lowest concentration that inhibited microbial growth was taken as the MIC. From the 96-well plate, 10 µL of each concentration mixture was aspirated, spread on the plate, and incubated at a constant temperature for 24 to 48 h. The lowest concentration at which no colonies grew on the plate was taken as the minimum bactericidal concentration (MBC) [25].

## 3. Results and Discussion

### 3.1. LC-MS Analysis

**Figure 1:**
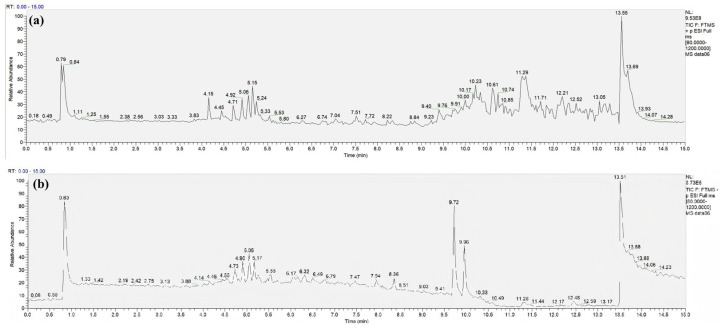
TIC diagrams. (**a**) Positive ion mode; (**b**) negative ion mode.

The black corncob extract was analyzed by LC-MS, and a total ion current chromatogram (TIC) of the sample was obtained. As shown in Figure 1, peaks from the sample were abundant, and characteristic chromatographic peaks were detected in different retention time periods, indicating that the black corncob extract contains multiple polar and moderately polar active components.

Based on the analysis by the Compound Discoverer 3.3 software, 25 compounds were identified as shown in Table 2. The LC-MS analysis indicated that the black corncob extract contained a variety of natural bioactive components, mainly belonging to the three categories of phenolic acids, flavonoids, and anthocyanins. Among the phenolic acid components, chlorogenic acid and ferulic acid were identified; the flavonoids were represented by quercetin and rutin; in addition, various anthocyanin substances such as cyanidin-3-O-glucoside, peonidin-3-O-glucoside, and pelargonidin-3-O-glucoside were successfully detected.

### 3.2. Optimization of the Synthesis Process of BC-AuNPs

The reduction of Au^3+^ by black corncob extract was confirmed by the formation of a unique wine-red color in the solution, attributed to the small size of the gold nanoparticles, which was caused by the plasmon resonance absorption. When the free electrons on the surface of gold nanoparticles are excited by an incident optical field to undergo collective resonant oscillations, a unique optical phenomenon known as surface plasmon resonance (SPR) is generated, leading to intense light absorption at specific wavelengths and an obvious color change in the solution (Supplementary Materials, Figures S1–S4) [26]. UV-Vis is currently one of the most widely used techniques for detecting the formation, size and stability of gold nanoparticles in water-soluble colloidal solutions: the maximum absorption wavelength of the transverse stretching vibration peak corresponds to the color change (with a darker color indicating a larger maximum absorption wavelength), and the half-peak width corresponds to the distribution of gold nanoparticles (with a narrower width indicating a more uniform distribution) [27]. When the synthesized gold nanoparticles are spherical and their size is less than 60 nm, the characteristic SPR wavelength band is usually around 500–550 nm [28]. Therefore, this study conducted selection and optimization of the synthesis process of BC-AuNPs based on UV-Vis spectroscopy.

**Figure 2:**
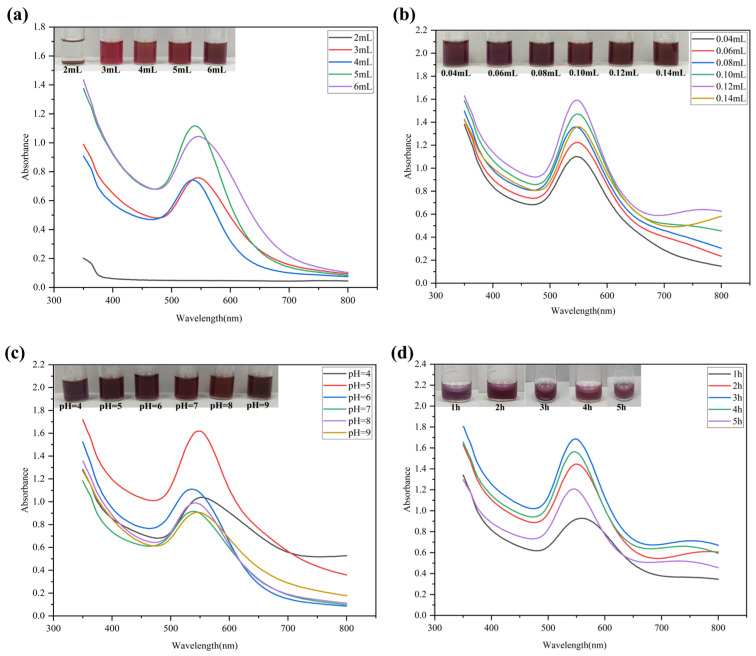
Research on factors affecting the preparation of BC-AuNPs. (**a**) Volume of black corncob extract added; (**b**) volume of 3% HAuCl_4_ added; (**c**) pH; (**d**) reaction time.

(1) The volume change in black corncob extract: As shown in the Figure 2a UV-Vis spectra, when the volume of the black corncob extract was 2 mL, no absorption peaks were observed in the range of 500–550 nm, indicating that no gold nanoparticles were formed. As the volume increased from 3 mL to 5 mL, a gradual increase in peak intensity and an improvement in peak symmetry were observed, suggesting an enhanced formation of BC-AuNPs. Notably, at a volume of 5 mL, the characteristic peak exhibited a strong response and a relatively narrow bandwidth. When the volume increased to 6 mL, the characteristic peak shifted towards the red, and the response and symmetry weakened. The results showed that appropriately increasing the volume of the black corncob extract could promote the formation of gold nanoparticles, while excessive addition led to the extraction substance adsorbing onto the surface of the formed product, causing agglomeration and hindering the uniform synthesis. Therefore, 5 mL was the optimal volume for extracting the black corncob extract.
(2) The volume change of 3% HAuCl_4_: As shown in the Figure 2b UV-Vis spectra, when the volume of 3% HAuCl_4_ solution increased from 0.04 mL to 0.12 mL, the response of the characteristic peaks and their symmetry gradually strengthened, but no significant displacement change occurred. When the volume was further increased to 0.14 mL, no significant shift in the characteristic peaks was observed, but the response weakened and the spectral bands broadened, indicating that the excessive HAuCl_4_ solution was not conducive to uniform synthesis. The results showed that the most suitable volume of the 3% HAuCl_4_ solution was 0.12 mL.
(3) Optimization of pH conditions: This experiment was conducted by using a highly diluted NH_3_·H_2_O solution for adjustment. As shown in the Figure 2c UV-Vis spectra, when the pH value ranged from 4 to 5, the characteristic peak showed a strong response and the spectral band was relatively narrow, indicating that the size uniformity of the generated gold nanoparticles was good. However, when the pH increased from 6 to 9, the characteristic peaks showed a blue shift, but the response and symmetry significantly weakened. This was because reducing groups such as polyphenols and flavonoids in the black corncob extract deprotonated, strengthening intermolecular electrostatic repulsion. The stability of the gold nanoparticles was disrupted, leading to partial aggregation. Therefore, pH = 5 was chosen as the optimal pH value for the reaction.
(4) Reaction time: Figure 2d shows the UV-Vis spectra of BC-AuNPs prepared at different reaction times. As the reaction time increased, the response value gradually increased and the peak shape became more symmetrical. When the reaction time reached 3 h, the response of the characteristic peak reached the maximum value. As the reaction time was extended, the response value of the characteristic peak decreased, the peak width increased, and the particle distribution became uneven. After comparison, it was concluded that the optimal reaction time was 3 h.

### 3.3. Structural Characterization of BC-AuNPs

#### 3.3.1. UV-Vis Spectroscopic Analysis

**Figure 3:**
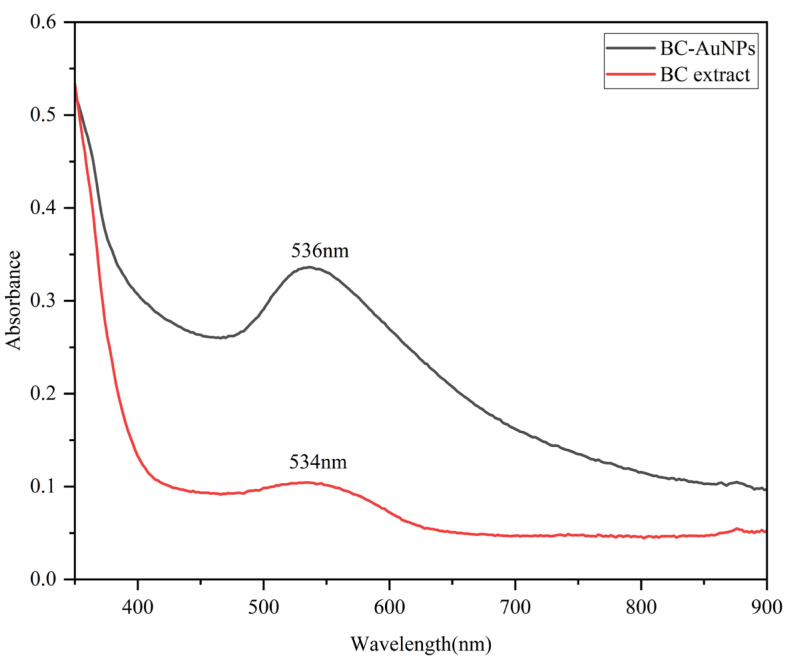
The UV-Vis absorption spectra of BC-AuNPs and the BC extract.

Figure 3 shows the UV-Vis spectra of the BC-AuNP composite material and the black corncob extract. The BC-AuNP composite material exhibited a characteristic sharp and symmetrical peak shape at 536 nm. The results fall within the characteristic SPR peak range for gold nanoparticles and most of the nanoparticles were spherical [29]. Meanwhile, the black corncob extract showed a weak absorption peak at 534 nm. This was because the black corncob extract contained anthocyanin pigments, and anthocyanins have strong absorption peaks between 500 and 540 nm [30].

#### 3.3.2. FTIR Analysis

**Figure 4:**
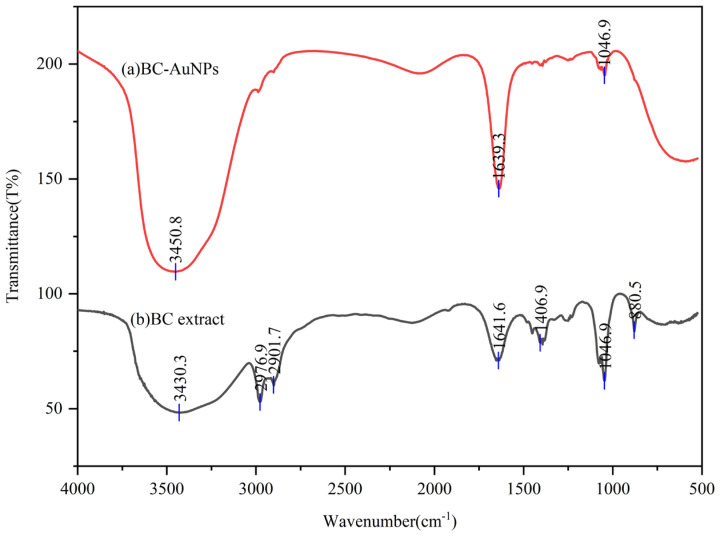
FTIR spectra of BC-AuNPs and BC extract.

The FTIR spectra of the BC-AuNP composite material and the black corncob extract within the range of 500–4000 cm^−1^ are shown in Figure 4. In the FTIR spectrum of the BC-AuNP composite material shown in Figure 4(a), the broad absorption peak at 3450.8 cm^−1^ was due to the intermolecular hydrogen bond stretching vibration formed by the hydroxyl group O–H [31]. The sharp absorption peak at 1639.3 cm^−1^ was related to the stretching vibration of the aromatic ring of C=C, which was consistent with the characteristic peak of the aromatic ring in anthocyanins and polyphenols; the absorption peak at 1046.9 cm^−1^ indicated the stretching vibration of the C–O single bond [32]. In Figure 4(b), the characteristic absorption bands of the FTIR spectrum of the black corncob extract were very similar to those of the BC-AuNP composite material. Among them, the broad peak at 3430.3 cm^−1^ mainly originated from the stretching vibration of the phenolic hydroxyl groups in polyphenols. Compared with the BC-AuNPs, the O–H stretching vibration peak of the extract was broader, indicating the presence of a stronger and wider intermolecular hydrogen bond network. This proved that these hydroxyl groups were involved in the reduction of Au^3+^ and the surface stabilization of Au^0^, resulting in a reduction of hydrogen bond donors and weakening of the intermolecular hydrogen bond network. The characteristic peaks at 2976.9 cm^−1^ and 2901.7 cm^−1^ belong to the C–H stretching vibration of alkanes. The peak at 1406.9 cm^−1^ mainly belonged to the bending vibration of the methyl group, while the absorption band at 880.5 cm^−1^ corresponds to the C–H bending vibration of the aromatic ring. The absorption peak at 1641.6 cm^−1^ originated from the stretching vibration of the aromatic C=C ring; the absorption peak at 1046.9 cm^−1^ represented the stretching vibration of the C–O single bond [33]. These results confirmed that the active components in the black corncob extract were involved in the reduction of Au^3+^ and were associated with the formation of gold nanoparticles.

**Figure 5:**
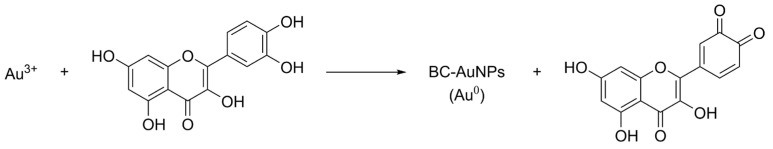
Chemical reaction mechanism of BC-AuNPs.

Based on the LC-MS analysis results and FTIR spectral data, this study confirmed that the synthesis mechanism of the gold nanoparticles (AuNPs) is mediated by the black corncob extract. According to LC-MS analysis, the extract from black corncob contains abundant flavonoid components, and quercetin is a representative active substance. As shown in Figure 5, quercetin (3,3′,4,5,7-pentahydroxyflavone) contains multiple hydroxyl groups and exhibits excellent reducing properties [34]. Its 1,2-benzenediol structure donates electrons, reducing Au^3+^ to Au^0^ and itself oxidizing to the 1,2-benzoquinone structure [35]. The Au^0^ gold atoms formed continuously aggregate to form nano-sized gold nuclei and gradually grow into spherical AuNPs. Oxidized quercetin quinone derivatives serve as stabilizers. Through hydrogen bonds, π–π stacking, and coordination between carbonyl groups and the gold surface, they adsorb onto the surface of AuNPs, forming a coating layer that increases the steric hindrance [36,37]. Meanwhile, under acidic conditions, the quercetin quinone derivatives have a positively charged surface. Through electrostatic repulsion, they prevent the aggregation of gold nanoparticles, thereby ensuring the stability of the system [38].

#### 3.3.3. TEM and HRTEM Analysis

**Figure 6:**
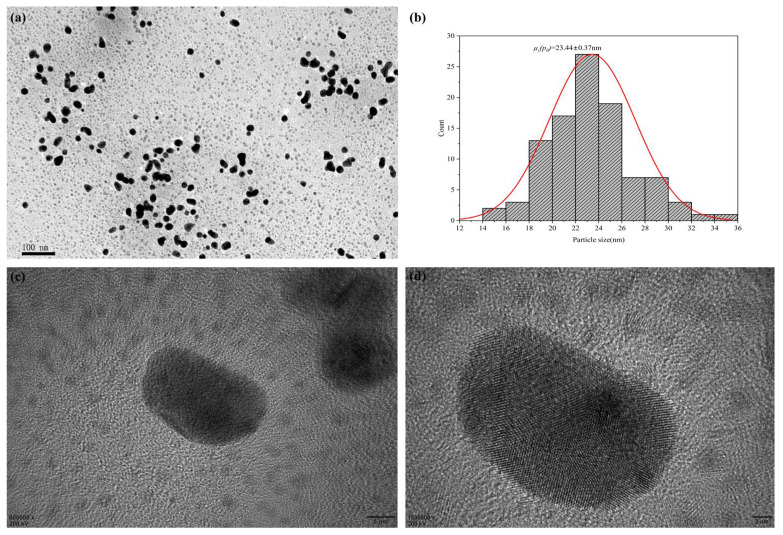
The TEM and HRTEM results for the BC-AuNPs. (**a**) Typical TEM image; (**b**) particle size distribution (*n* = 100, average particle size = 23.44 ± 0.37 nm); (**c**,**d**) HRTEM images.

The transmission electron microscopy scans of the BC-AuNP composite material are shown in Figure 6. As shown in (a), a typical TEM image was produced, in which most of the synthesized BC-AuNP composite materials had good dispersion, and the particle shapes were mainly spherical; as shown in (b), the particle size distribution graph showed that they conformed to a normal distribution. The average particle size was 23.44 ± 0.37 nm (*n* = 100), indicating that the sizes of the synthesized BC-AuNP particles were extremely uniform. As shown in (c) and (d), HRTEM clearly showed that the BC-AuNPs were spherical or ellipsoid particles with clear boundaries and uniform sizes, ranging from 20 to 30 nm. Furthermore, the BC-AuNPs presented a crystalline state and had a polycrystalline domain structure. However, due to the background contrast of the carbon substrate, the organic coating layer on the particle surface could not be clearly observed [39].

#### 3.3.4. SEM Analysis

**Figure 7:**
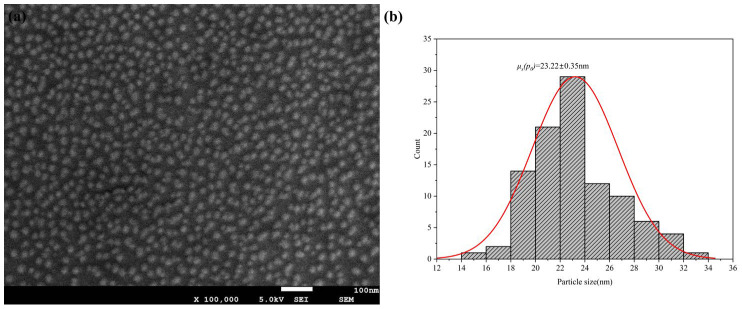
The SEM results for the BC-AuNPs. (**a**) Typical SEM image; (**b**) particle size distribution (*n* = 100, average particle size = 23.22 ± 0.35 nm).

The scanning electron microscopy analyses of the BC-AuNP composite material are shown in Figure 7. As shown in (a), a typical scanning electron microscopy image was obtained, in which it can be observed that most of the synthesized BC-AuNP composite materials were monodisperse spherical nanoparticles with uniform particle sizes. As shown in (b), the particle size distribution graph was in accordance with a normal distribution. The average particle size was 23.22 ± 0.35 nm (*n* = 100), which was consistent with the TEM particle size distribution graph.

#### 3.3.5. DLS and Z-Potential Analysis

**Figure 8:**
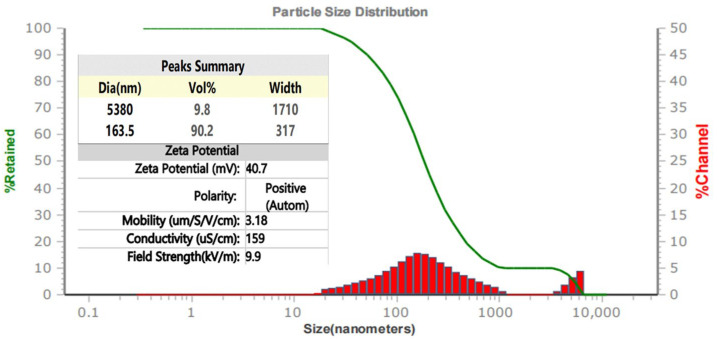
The DLS and Z-potential results for the BC-AuNPs.

As shown in Figure 8, DLS indicated that the hydrodynamic size of most BC-AuNPs was approximately 163.5 nm, which was much larger than the particle size estimated by TEM because the fluid dynamics size determination referred to the size of BC-AuNPs in the hydrated state. Besides the gold core itself, the measurement also included the hydrated layer, the organic coating layer, and other compounds that may stabilize the nanoparticles [40]. In addition, the DLS display system also shows that 9.8% of the signals had extremely large particle sizes. It was speculated that a small number of gold nanoparticles had undergone irreversible aggregation. However, since the proportion of this extremely large aggregated component was extremely low, it indicated that the prepared BC-AuNPs still possessed good dispersibility and stability. The Z-potential was an important indicator for measuring the surface charge and stability of the nanoparticles. Generally, an absolute value of the Z-potential of the nanoparticles greater than 30 mV indicates good stability [41,42]. As shown in Figure 8, the Z-potential of the prepared BC-AuNPs was 40.7 mV, indicating that the stability of the BC-AuNPs was good.

### 3.4. Study of Photothermal Properties

**Figure 9:**
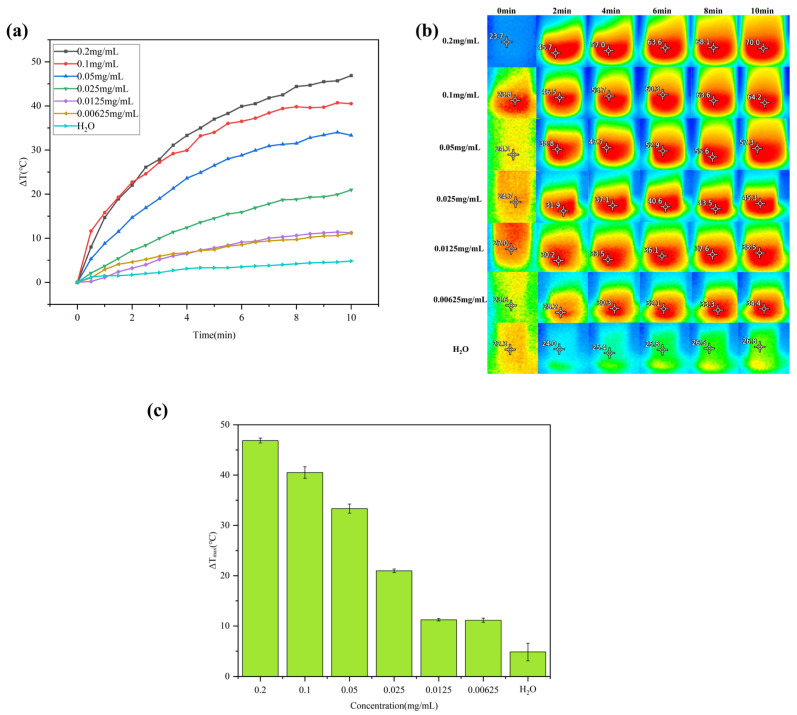
Photothermal property plot for the BC-AuNPs. (**a**) Temperature changes of different concentrations of BC-AuNPs and ultra-pure water under 808 nm laser irradiation for 10 min; (**b**) photothermal images of different concentrations and durations; (**c**) the maximum temperature difference variation and error distribution of the photothermal experiments at various concentration gradients.

As shown in the temperature change graph in Figure 9a, the different concentrations of BC-AuNP composite materials all showed a significant increase in temperature compared to pure water, and the temperature increase effect was positively correlated with their concentrations. From the infrared thermal images (b), it can be observed that as the laser irradiation time increased, the central part of the BC-AuNP composite material heated up significantly, and the heating amplitude showed a concentration-dependent pattern. For the BC-AuNP composites with concentrations of 0.00625 mg/mL, 0.0125 mg/mL, 0.025 mg/mL, 0.05 mg/mL, 0.1 mg/mL and 0.2 mg/mL, after 10 min of 808 nm laser irradiation, their final temperatures reached 34.4 °C, 38.5 °C, 45.3 °C, 57.3 °C, 64.2 °C and 70.0 °C, respectively; in contrast, no significant temperature increase was observed in ultra-pure water under the same conditions. Therefore, the BC-AuNP composite material absorbed light energy under irradiation with an 808 nm laser and converted it into thermal energy, increasing the temperature, making it a good photothermal material.

### 3.5. Study of Photothermal Cycling Performance

**Figure 10:**
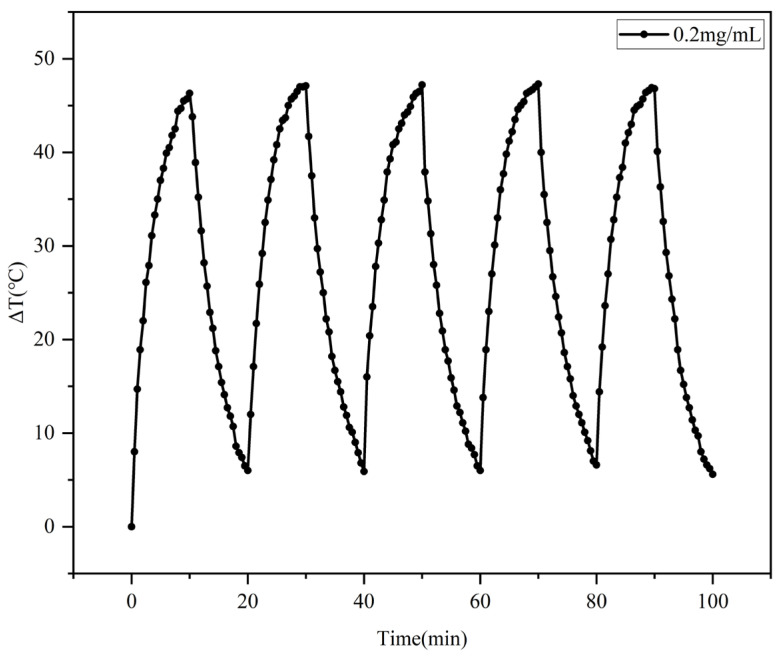
Photothermal cycling curve of the 0.2 mg/mL BC-AuNPs.

The photothermal cycling curve of the 0.2 mg/mL BC-AuNP composite material is shown in Figure 10. Under the irradiation of an 808 nm laser, the peak temperature and response time of this material remained stable, and it exhibited good reproducibility in multiple cycles. After 10 min of exposure, the temperature increase was similar in both cases. Then, they began to cool down for another 10 min, and the temperature decrease was basically the same. By calculating the standard deviation of the temperatures from five parallel experiments through mathematics, the standard deviation of the temperature after 10 min of heating was 0.404 °C, and the standard deviation after 10 min of cooling was 0.363 °C. The calculation results showed that the temperature standard deviation was less than 1 °C, indicating a very small value. This indicated that the results of the five consecutive experiments were mostly consistent, and the photothermal stability performance was good. There would be no significant drop in temperature during repeated experiments.

### 3.6. Study of Photothermal Conversion Efficiency

**Figure 11:**
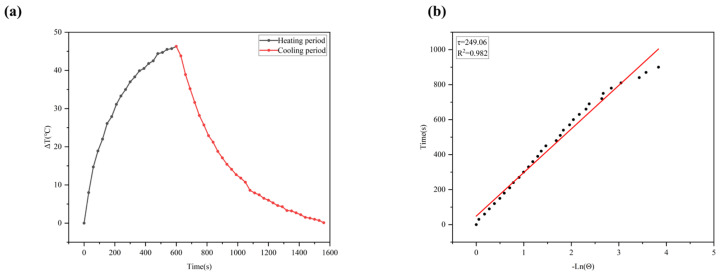
Photothermal conversion efficiency curve for the 0.2 mg/mL BC-AuNPs. (**a**) Photothermal heating and cooling curve; (**b**) plot of ln(Θ) vs. cooling time.

Figure 11a shows the photothermal heating and cooling curve of the 0.2 mg/mL BC-AuNP composite material. The temperature of this material significantly increased with an increase in irradiation time, indicating a stable photothermal conversion capability. Figure 11b shows a graph of the natural logarithmic relationship between the cooling time and temperature for the 0.2 mg/mL BC-AuNP composite material. Based on the data in the graph, τ = 249.06, R^2^ = 0.982. Using the above formula, η = 41.3%.

### 3.7. Antibacterial Activity Study

**Figure 12:**
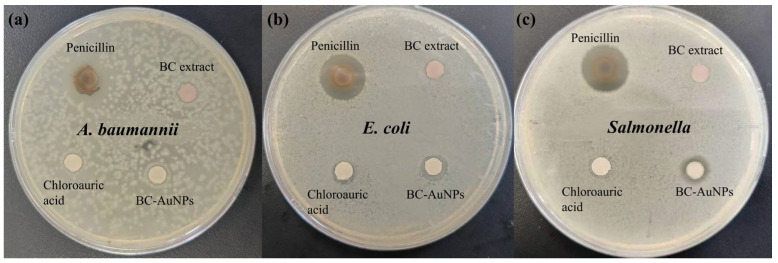
Results of inhibition-zone experiments with BC-AuNPs (**a**) *A. baumannii*; (**b**) *E. coli*; (**c**) *Salmonella*.

As shown in Figure 12, the antibacterial effect of 0.01% penicillin solution on *E. coli* and *Salmonella* was the strongest. The 0.2 mg/mL BC-AuNP solution showed some antibacterial activity, while the black corncob extract and 3% HAuCl_4_ solution had no obvious antibacterial effect. However, neither the 0.01% penicillin solution nor the 0.2 mg/mL black corncob–AuNP solution showed any activity against *A. baumannii*. The reason lies in the fact that *A. baumannii* is a multidrug-resistant strain, and the 0.01% penicillin solution is not effective on its own. Moreover, the diffusion ability of gold nanomaterials in agar was limited, making it difficult for them to reach an effective bacteriostatic concentration [43,44]. In addition, as shown in Table 3, the MIC of BC-AuNPs against *E. coli* was 25.00 µg/mL and against *Salmonella* it was 50.00 µg/mL.

The *E. coli* and *Salmonella* used in this experiment are both Gram-negative bacteria. The cell walls of Gram-negative bacteria are negatively charged and consist of a thin peptidoglycan layer, with an additional outer membrane composed of lipopolysaccharides [45]. BC-AuNPs can release gold ions, such as Au^+^ and Au^3+^ [46]. Research has shown that the released gold ions possess superior toxicity. They adhere to and damage the cytoplasmic membrane, interact with functional groups of proteins and nucleic acids, such as –SH, –COOH, and –NH, alter the spatial conformation of enzymes, lose their activity, and thereby inhibit microorganisms [47]. The smaller the particle size of AuNPs, the larger their surface area, which leads to a faster release of Au^+^ and Au^3+^ to get a better antibacterial effect [48]. In addition, the BC-AuNPs themselves also have an electrostatic attraction effect on the bacterial cell wall, causing a decrease in membrane potential, cell membrane damage, and bacterial death [49].

In addition, the surface of the prepared gold nanoparticles has a synergistic effect with the phenolic acids, flavonoids, anthocyanins, and other active components in the black corncob extract, forming a stable natural biocompatible coating. These functional stabilizers can specifically bind to bacterial surface receptors through non-covalent bonds such as hydrogen bonds, hydrophobic interactions, and π–π stacking, thereby disrupting the membrane structure and inhibiting the activity of metabolic enzymes. This enhances the targeting ability and antibacterial activity of BC-AuNPs [50,51].

However, the structure of *A. baumannii* differs from that of *E. coli* and *Salmonella*. The outer membrane of *A. baumannii* is an ultra-dense lipopolysaccharide layer, and the lipid phosphate groups are extensively modified by cations, reducing the negative charge on the membrane surface [52,53]. Research has shown that the ultra-dense lipid polysaccharide layer can physically isolate the contact of BC-AuNPs, forming a physical barrier for larger-sized (>20 nm) nanoparticles, preventing damage to the cell wall and cytoplasmic membrane [54,55]. Moreover, the outer membrane of *A. baumannii* has low permeability and can chelate metal ions, thereby reducing the toxicity of the released gold ions [56,57]. Furthermore, the outer membrane of *A. baumannii* lacks specific receptors, which will affect the targeting ability of BC-AuNPs and prevent them from exerting antibacterial effects [58].

### 3.8. Comparison with Previous Studies

As shown in Table 4, the performance of the BC-AuNPs is compared with other reported green synthesis systems for gold nanoparticles. The advantages of this research were further verified. This study used black corncob from agricultural waste as the raw material to investigate the influence of the active component quercetin in the raw material on the synthesis of gold nanoparticles. In terms of particle size, the as-prepared BC-AuNPs were smaller, and the distribution was more uniform. According to synthesis efficiency, both this study and previous studies adopted the green synthesis process, which was carried out under mild conditions and had comparable efficiency levels. Furthermore, for common pathogenic bacterium such as *E. coli*, the MIC values in previous studies were usually greater than 100 µg/mL. However, the antibacterial performance of the BC-AuNPs prepared in this study was superior to that reported in the previous literature, reaching 25.00 µg/mL [59,60,61].

## 4. Conclusions

This study employed a novel method for the green synthesis of AuNPs from black corncob extract with excellent properties. The black corncob extract contained phenolic acids, flavonoids, and anthocyanins, determined by LC-MS analysis. The optimal reaction conditions for the BC-AuNPs were determined through single-factor experiments. The BC-AuNPs were further characterized by UV-Vis, FTIR, TEM, HRTEM, SEM, DLS, and Z-potential measurement. The BC-AuNPs were spherical particles with good uniformity and dispersion, and the average diameter was 23.0 nm. The photothermal experiments demonstrated that the BC-AuNPs exhibit excellent photothermal conversion and cycling performance, with a photothermal conversion efficiency of up to 41.3%. The antibacterial test results showed that this material has a good antibacterial effect against *E. coli* and *Salmonella*. Overall, the BC- AuNPs have potential to be developed as a new type of safe antibacterial and photothermal therapy material.

## Figures and Tables

**Table 1:** Synthesis of BC-AuNPs using single-factor experiments.

Sample	Volume of Black Corncob Extract (mL)	Volume of 3% HAuCl_4_ (mL)	pH	Reaction Time (h)	Temperature (°C)
1	2	0.12	5	3	50
2	3	0.12	5	3	50
3	4	0.12	5	3	50
4	5	0.12	5	3	50
5	6	0.12	5	3	50
6	5	0.04	5	3	50
7	5	0.06	5	3	50
8	5	0.08	5	3	50
9	5	0.10	5	3	50
10	5	0.12	5	3	50
11	5	0.14	5	3	50
12	5	0.12	4	3	50
13	5	0.12	5	3	50
14	5	0.12	6	3	50
15	5	0.12	7	3	50
16	5	0.12	8	3	50
17	5	0.12	9	3	50
18	5	0.12	5	1	50
19	5	0.12	5	2	50
20	5	0.12	5	3	50
21	5	0.12	5	4	50
22	5	0.12	5	5	50

**Table 2:** Identification of 25 compounds via the Compound Discoverer software.

No.	RT (min)	Additive ion	*m*/*z*	Molecular Weight	Error (ppm)	Molecular Formula	Compound Name
**1**	9.726	[M + H − H_2_O]^+1^	439.35607	456.35905	−2.78	C_30_H_48_O_3_	Oleanolic acid
**2**	4.586	[M + H]^+1^	610.14130	302.04234	−1.28	C_27_H_30_O_16_	Rutin
**3**	6.648	[M − H + HAc]^−1^	193.05829	134.03546	−1.71	C_10_H_10_O_4_	Ferulic acid
**4**	4.842	[M + Na]^+1^	377.08597	354.0942	−2.13	C_16_H_18_O_9_	Chlorogenic acid
**5**	7.112	[M − H]^−1^	301.10280	302.04205	−2.03	C_15_H_10_O_7_	Quercetin
**6**	4.487	[M + H]^+1^	449.10689	448.10023	−0.69	C_21_H_20_O_11_	Kuromanin
**7**	5.741	[M − H]^−1^	463.07528	464.09439	−2.29	C_21_H_20_O_12_	Quercetin-3β-D-glucoside
**8**	4.735	[M – H − H_2_O]^−1^	447.10123	466.11009	0.90	C_21_H_20_O_11_	Astragalin
**9**	4.887	[M + H]^+1^	285.04014	286.05120	−1.09	C_15_H_10_O_6_	Kaempferol
**10**	10.042	[M − H]^−1^	487.34162	488.34886	−2.80	C_30_H_48_O_5_	Asiatic acid
**11**	9.619	[M + H]^+1^	279.23238	278.22443	−0.44	C_18_H_30_O_2_	α-Linolenic acid
**12**	0.737	[M + H]^+1^	153.12824	152.11989	−1.51	C_10_H_16_O	D-(+)-Camphor
**13**	8.293	[M + H]^+1^	322.27093	321.2636	3.81	C_20_H_32_O_2_	Arachidonic acid
**14**	12.293	[M − H]^−1^	339.32573	340.33325	−2.62	C_22_H_44_O_2_	Docosanoic acid
**15**	9.732	[M + H − H_2_O]^+1^	161.05940	178.06286	−0.82	C_10_H_10_O_3_	3-Methoxycinnamic acid
**16**	4.510	[M + H]^+1^	287.05303	286.04686	−3.03	C_15_H_10_O_6_	Maritimetin
**17**	5.134	[M + H]^+1^	303.04873	302.04241	−0.76	C_15_H_10_O_7_	Bracteatin
**18**	4.907	[M + H]^+1^	303.05106	304.05827	−0.15	C_15_H_12_O_7_	Nigrescin
**19**	14.971	[M − H]^−1^	459.13133	460.13655	−0.82	C_23_H_24_O_10_	7-Hydroxy-5,6-dimethoxyflavone 7-glucoside
**20**	5.518	[M − H]^−1^	435.13031	436.13658	−0.82	C_21_H_24_O_10_	Nothofagin
**21**	5.831	[M − H]^−1^	299.01873	300.02693	−0.26	C_15_H_8_O_7_	Demethylwedelolactone
**22**	4.993	[M + H]^+1^	401.15895	400.15175	−1.17	C_22_H_24_O_7_	Melafolone
**23**	0.929	[M − H]^−1^	361.09397	362.1013	3.09	C_18_H_18_O_8_	5,7,3′-Trihydroxy-6,4′,5′-trimethoxyflavanone
**24**	1.141	[M + H]^+1^	551.15525	550.1479	0.65	C_29_H_26_O_11_	Formononetin 7-O-(2″-p-hydroxybenzoylglucoside)
**25**	13.556	[M − H]^−1^	593.15432	594.16078	3.81	C_27_H_30_O_15_	Palasitrin

**Table 3:** The MIC and MBC results of penicillin solution and BC-AuNPs (μg/mL).

	*E. coli*		*Salmonella*	
	MIC	MBC	MIC	MBC
Penicillin solution	6.25	12.50	6.25	12.50
BC-AuNPs	25.00	50.00	50.00	100.00

**Table 4:** Comparative analysis with previously reported green-synthesized AuNP systems.

Synthetic Material	Particle Size	Synthesis Condition	MIC of *E. coli* (μg/mL)	Study (Year)
*Cassia alata* leaves extract	63.647 ± 1.334 nm	Microwave-assisted	500	(Situmorang et al., 2025) [59]
*Salvia sclarea* L. extract	70–80 nm	At room temperature	104.17	(Zarei et al., 2025) [60]
*Lyngbya confervoides* extract	4–22 nm	At room temperature	100	(Swain et al., 2025) [61]
Black corncob extract	23.0 nm	50 °C in a water bath	25.00	This study

## Data Availability

The data are available from the corresponding author upon reasonable request.

## Supplementary Materials

The following supporting information can be downloaded at: https://www.mdpi.com/article/10.3390/nano16110646/s1. Figure S1: Photographs of BC-AuNPs synthesized by varying black corncob extract volumes; Figure S2: Photographs of BC-AuNPs synthesized by varying 3% HAuCl_4_ volumes; Figure S3: Photographs of BC-AuNPs synthesized under different pH conditions; Figure S4: Photographs of BC-AuNPs synthesized by different reaction times.

## Abbreviations

The following abbreviations are used in this manuscript:

BC	Black corncob
BC-AuNPs	Black corncob–gold nanoparticles
UV-Vis	Ultraviolet–visible
FTIR	Fourier-transform infrared
TEM	Transmission electron microscopy
HRTEM	High-resolution transmission electron microscopy
SEM	Scanning electron microscope
DLS	Dynamic light scattering
Z-potential	Zeta potential
MIC	Minimum inhibitory concentrations
AMR	Antimicrobial resistance
LC-MS	Liquid chromatography–mass spectrometry
MS	Mass spectrometry
MS_2_	Secondary mass spectrometry
MBC	Minimum bactericidal concentration
TIC	Ion current chromatogram
SPR	Surface plasmon resonance
AuNPs	Gold nanoparticles
