# Supplementary material for: Green Synthesis of Gold Nanoparticles with Good Photothermal Properties and Antibacterial Activity from Black Corncob Extract
Source: Nanomaterials (Basel). 2026 May 22;16(11):646. doi: 10.3390/nano16110646 (PMC13258648; doi:10.3390/nano16110646)
Supplement: Supplementary file 1 [file nanomaterials-16-00646-s001.zip › nanomaterials-4276851-supplementary.pdf]

Supplementary Information

Green Synthesis of Gold Nanoparticles with Good Photothermal Properties and Antibacterial Activity from Black Corncob Extract

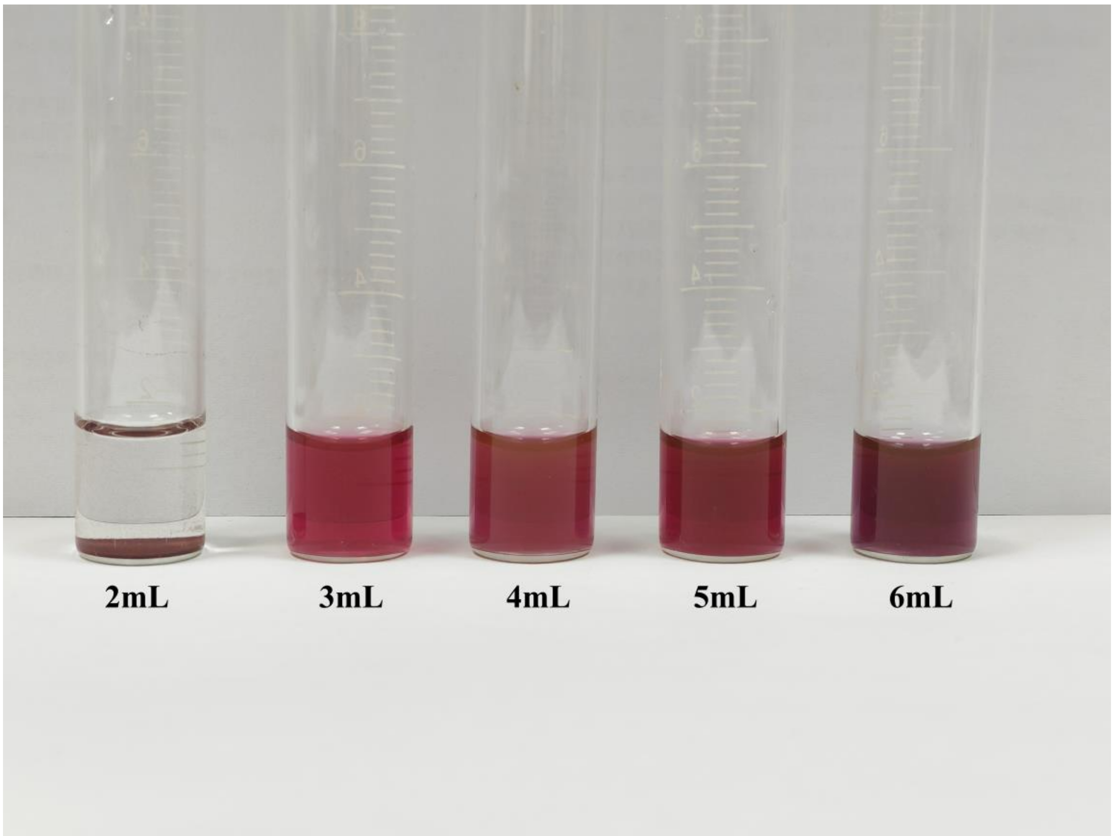

Figure S1. Photographs of BC-AuNPs synthesized by varying black corncob extract volumes

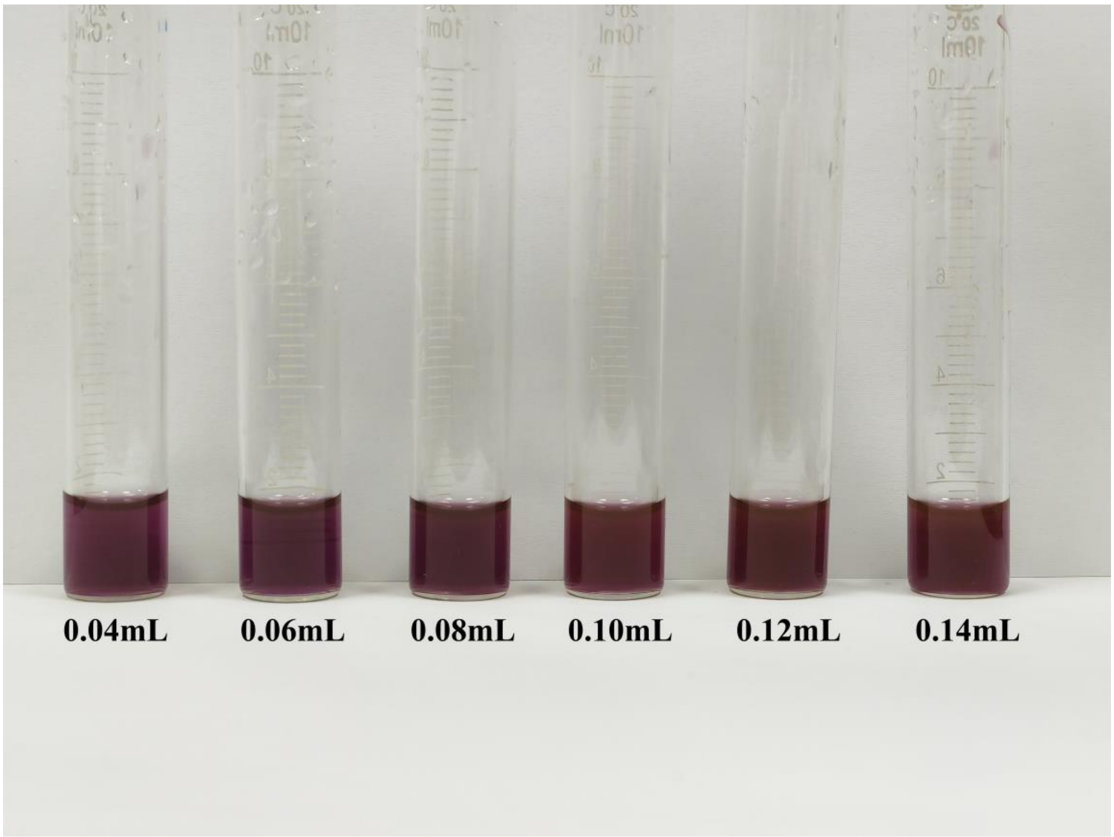

Figure S2. Photographs of BC-AuNPs synthesized by varying 3% $HAuCl_4$ volumes

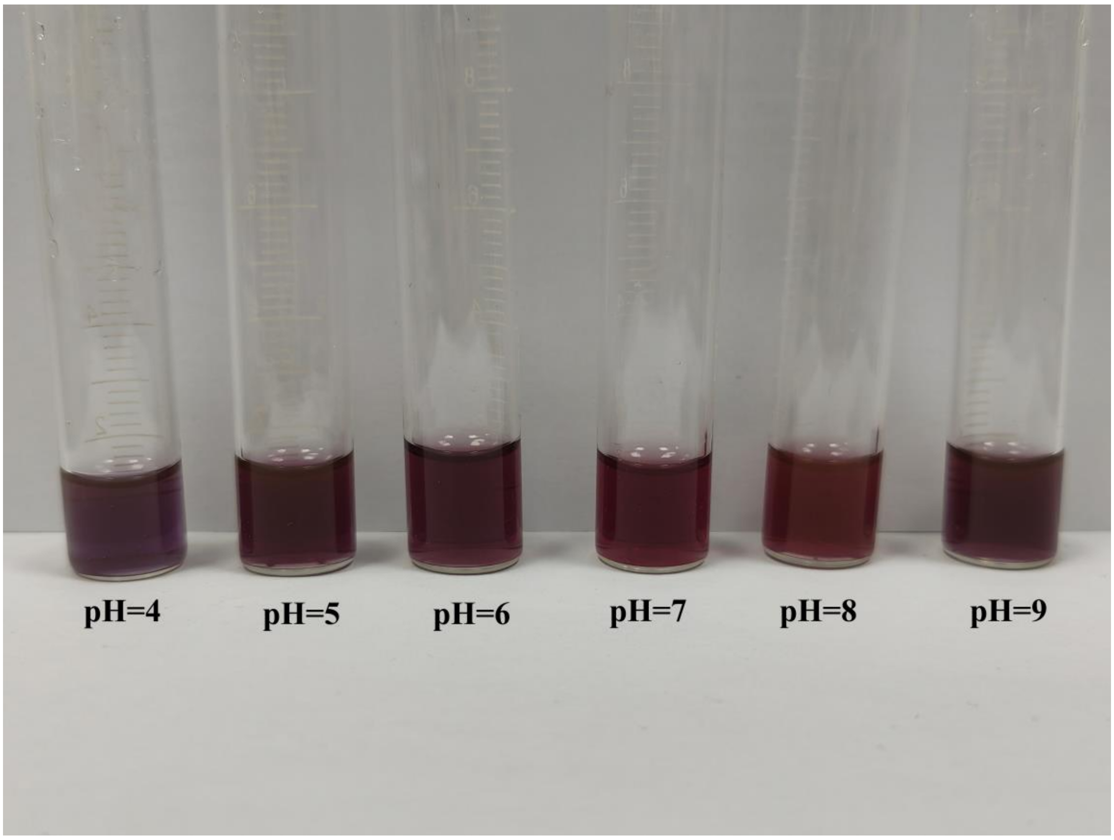

Figure S3. Photographs of BC-AuNPs synthesized under different pH conditions

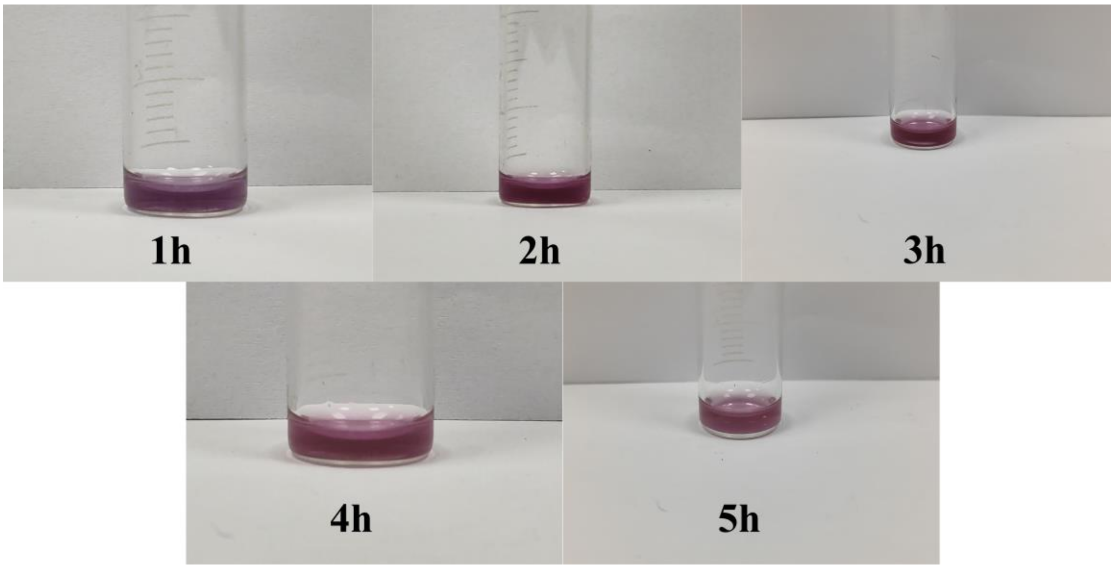

Figure S4. Photographs of BC-AuNPs synthesized by different reaction times
